# Improving the management of chronic non-cancer pain in primary care: Results of a multifaceted quality improvement initiative

**DOI:** 10.1186/1748-5908-10-S1-A45

**Published:** 2015-08-20

**Authors:** Daren Anderson, Ianita Zlateva, Brent Moore

**Affiliations:** 1 Weitzman Institute, Community Health Center Inc, Middletown, CT 06457 USA; 2 Department of Psychiatry, Yale University School of Medicine, New Haven, CT 06510 USA

## 

How the research advances dissemination and implementation research: The management of chronic pain poses a significant challenge for primary care practices, particularly those caring for medically underserved patient populations. Experience in the Veterans Health Administration (VHA) has demonstrated that implementation of a Stepped Care Model for Pain Management (SCM-PM) can improve outcomes for patients with chronic pain. In this project, we advance the dissemination and implementation research by describing the use of the Promoting Action on Research Implementation in Health Services (PARIHS) Framework to guide the adaptation, implementation, and dissemination of the SCM-PM to a non-VHA setting-a large, statewide Federally Qualified Health Center.

## Overall project implementation

Chronic pain is extremely common in safety-net practices. The Stepped Care Model for Pain Management (SCM-PM) has been shown to improve outcomes in the Veterans Health Administration (VHA) system. To examine whether this model is transferable to non-VHA settings we undertook a three-year quality improvement project to adapt and implement the SCM-PM in a large, statewide Federally Qualified Health Center.

This project used an observational mixed methods evaluation framework and used the PARIHS Framework to guide the design of the intervention. Study subjects included all patients and providers (PCPs) of the health center. The principal goals of the project were 1) to improve the screening for and management of routine pain complaints in primary care using basic tools and protocols to improve the assessment, documentation, treatment, and monitoring of pain; and 2) to provide additional resources and supports for PCPs to assist in managing more complex cases.

The use of opioid treatment agreements among patients using opioids chronically (COT), increased from 49% to 64%. The number of COT patients with a urine drug test within the preceding six months increased from 867 (66%) to 1,097 (86%). Patients with a completed pain functional assessment increased from 428 (33%) to 589 (46%). The percentage of patients co-managed by an onsite behavioral health provider increased from 22.5% to 24.4%. Referrals to chiropractors also increased. There was a decline in number of patients with pain receiving any opioid prescriptions from 43% to 40%, and in those receiving COT from 17.5% to 15.9%. The percentage of patients with an episode of severe pain decreased from 74% to 61%. Surveys showed that CHCI PCPs expressed increased confidence in their ability to manage pain effectively and had an increase of 9.2% in pain management knowledge scores.

## Identifying patients with chronic non-cancer pain in large datasets

To implement successfully the Stepped Care Model for Pain Management (SCM-PM) at our statewide Federally Qualified Health Center, we had to identify all patients with chronic non-cancer pain (CNCP). A straightforward method for identifying patients with CNCP solely using structured electronic health record (EHR) data does not exist. Individual data elements such as pain scores or diagnostic codes (ICD9) are not sufficiently reliable or comprehensive. Our objective was to develop and validate an accurate method to identify patients with CNCP using EHR data.

We identified patients with CNCP in our EHR system using a comprehensive set of data elements including diagnostic codes, patient-reported pain scores, and prescription opioid medications. Reviews of the medical chart were used to evaluate the accuracy of these data elements in all their combinations. Based on these evaluations we developed an algorithm to more accurately identify patients with CNCP. The algorithm's results were validated by comparing them with the documentation of chronic pain by the patient's treating clinician in 381 random patient charts.

The new algorithm, using pain scores, prescription medications, and ICD9 codes had a sensitivity and specificity of 84.8% and 97.7%, respectively. The algorithm was more accurate (95.0%) than pain scores (88.7%) or ICD9 codes (93.2%) alone. The Receiver Operating Characteristic was 0.981.

The newly developed and validated algorithm uses a combination of readily available elements from the EHR system to accurately identify patients with CNCP. By applying the algorithm to our patient population, we were able to gain a better understanding of the extent of chronic pain and how it is managed in our health centers. This helped us to better tailor our implementation efforts to the needs of the patients with CNCP and their primary care providers.

## Pain care quality documentation chart reviews

The Veterans Health Administration (VHA) has engaged in a system-wide transformational effort to implement the Stepped Care Model of Pain Management (SCM-PM) as its single standard of care for Veterans with painful conditions. Successful organizational improvement processes typically rely on reliable metrics to establish targets for improvement and to monitor progress. This project examined the utility of the measure of Pain Care Quality (PCQ) documentation in evaluating implementation of the SCM-PM at one VHA healthcare system and to explore its generalizability in a non-VHA Federally Qualified Health Center undergoing a similar organizational improvement effort.

From 2009 to 2012 a comprehensive pain management performance improvement approach was implemented at the VHA. Two hundred progress notes per year (July 2008 -June 2012) were randomly sampled from VHA primary care prescribers of chronic opioid therapy (COT). Using the PCQ extraction tool, each note was reviewed and coded for the presence of key dimensions of PCQ documentation, namely pain assessment, treatment, and reassessment of outcomes. General Estimating equations (GEE) controlling for provider and facility, with post-hoc pair comparisons were used to examine changes in PCQ items over the four years. This approach was replicated in a multi-site FQHC for two consecutive years (2011-2012).

Significant improvements were noted in pain reassessment and patient education, with trends in improvement noted for pain assessment and treatment planning. Several specific dimensions of pain assessment and treatment planning also improved significantly, including documentation of functional assessments (p < 0.001). Although post hoc comparisons generally documented improvements over time, some variability across the four years of observation suggest that these trends are not entirely linear. Although none of the dimensions of PCQ at the FQHC were significant, results suggest trends in a positive direction across all dimensions of PCQ.

